# B Cell Subset Analysis and Gene Expression Characterization in Mid-Luteal Endometrium

**DOI:** 10.3389/fcell.2021.709280

**Published:** 2021-08-10

**Authors:** Mengni Shen, Tim Child, Monica Mittal, Geet Sarodey, Rehan Salim, Ingrid Granne, Jennifer H. Southcombe

**Affiliations:** ^1^Nuffield Department of Women’s and Reproductive Health, L3 Women’s Centre, John Radcliffe Hospital, University of Oxford, Oxford, United Kingdom; ^2^Oxford Fertility, The Fertility Partnership, Oxford, United Kingdom; ^3^Department of Obstetrics and Gynaecology, Wolfson Fertility Center, St Mary’s and Hammersmith Hospitals, Imperial College Healthcare NHS Trust, London, United Kingdom

**Keywords:** endometrium, B cells, immune activation, Tfh cells, germinal center, IL-10

## Abstract

The human endometrium is the innermost mucosal membrane of the uterus and is the first point of contact for an implanting blastocyst. A wide variety of immune cells are found amongst the endometrial epithelial layers and stromal cells which both provide host immune responses against pathogens and also assist with placentation and pregnancy establishment, however, B cells have not been characterized, despite being a vital player in both adaptive and mucosal immunity. Through analysis of mid-luteal endometrial biopsies, we find 1–5% of endometrial immune cells are B cells, the majority were naïve or memory B cells, with few plasma cells. Compared with circulating B cells, endometrial B cells had an activated phenotype, with increased expression of CD69, HLA-DR, CD74, and CD83, and IL-10 production capacities. PD1^+^CXCR5^+^ICOS^+^ T follicular helper-like cells and FAS^+^IgD^–^BCL6^+^ germinal center B cells were also present in the endometrium, which may indicate that endometrial B cells are playing an active role through germinal center reactions in the human endometrial environment.

## Introduction

The human endometrium is a mucosal tissue containing immune cells which provide defense against uterine pathogens and have a role in immune tolerance for the successful establishment of pregnancy, as the semi-allogeneic embryo must invade through the tissue during placentation. NK, T, B, and macrophage cells are present in the endometrium, with reported changes to cellular proportions and functions across the menstrual cycle (Wira et al., 2015). The phenotypes and function of NK cells, T cells, and macrophages are widely characterized, with their dysregulation implicated in various reproductive pathologies such as infertility, recurrent pregnancy loss (RPL) and endometriosis (Brazdova et al., 2016; Vallvé-Juanico et al., 2019; Lucas et al., 2020). However, B cells are comparatively understudied in the endometrium despite their vital role in mucosal immunity (Vajdy, 2006).

Central to the adaptive humoral immune system, B cells display notable phenotypic and functional diversity between various known subsets. In brief, transitional B cells migrate from the bone marrow, subsequently mature into antigen-naïve B cells and enter into the periphery. After antigen encounter, naïve B cells can either differentiate into short-lived plasmablasts, or enter into germinal centers (GCs) and differentiate into memory B cells or long-lived plasma cells. This GC reaction relies on the involvement of T follicular helper (Tfh) cells and typically happens in the secondary lymphoid organs (SLOs), such as lymph nodes, tonsils, spleen or mucosal associated lymphoid tissue (MALT) (LeBien and Tedder, 2008). In addition to SLOs, GC or GC-like structures have also been described in ectopic or tertiary lymphoid structures (TLSs), where the GC supports local adaptive immune responses toward locally displayed antigens (Pipi et al., 2018). Other B cell subsets also exist, Breg are a rare B cell subset with regulatory/suppressor function, which is mostly but not solely achieved through the production of IL-10. Breg can also promote the development of Treg, a crucial player in the endometrial immune environment, possibly via TGF-β production (Lee et al., 2014). The involvement of Breg in pregnancy was recently recognized, in mice uteri, an expanded pool of IL-10-producing B cells are observed during implantation, contributing to the successful establishment of maternal immunological tolerance in early pregnancy through their regulatory function (Guzman-Genuino et al., 2019).

In this study, human endometrium and peripheral blood samples were collected from women during the mid-luteal phase of the menstrual cycle. We determined the B cell subsets present in the endometrium, specifically identifying a broad cellular repertoire, including the presence of both IL-10 producing B cells which may indicate the present of Breg, and also B cells that have a phenotype indicative of GC. We identify markers on endometrial B cells that are highly upregulated compared to peripheral blood B cells, which indicates endometrial B cells are likely tissue resident.

## Materials and Methods

### Sample Collection and Processing

The study was approved by the Oxford Research Ethics Committee C (ref:08/H0606/94 and ref:18/SC/0216). All participants gave written informed consent in accordance with the Helsinki Declaration of 1975. All recruits were patients attending Oxford Fertility, Wolfson Fertility Centre at Hammersmith Hospital, or the Recurrent Miscarriage Clinic in the Oxford University Hospitals NHS Foundation Trust, John Radcliffe Hospital between 2016 and 2018. All women were≤40 years of age. Blood and/or endometrial samples were taken in the mid-luteal phase of the menstrual cycle, during which the endometrial cells adapt for embryo receptivity, a period known as the window of implantation. LH-surge testing or known regular cycle length timing were used in determining the mid-luteal phase.

Peripheral blood mononuclear cells (PBMC) were isolated using lymphoprep (Axis Shield Diagnostics). Endometrial samples were obtained with an Endocell disposable endometrial cell sampler (Wallach Surgical devices, Trumbull, CT, United States). Endometrial samples were either digested with Collagenase D or mechanically digested if Collagenase D was shown to negatively impact the surface marker expression on the targeted cell type (i.e., CXCR5). For Collagenase D digestion, endometrial samples were incubated with 100 μg/ml DNase I and 2 mg/ml Collagenase D in Dulbecco’s Modified Eagle’s Medium + 10% Fetal bovine serum for 30 min in 37°C shaking incubator. For mechanical digestion, endometrial biopsies were cut into 1 mm^3^ fragments and transferred to 10 ml Dulbecco’s Modified Eagle’s Medium + 10% Fetal bovine serum with 100 μg/ml DNase I for mechanically digestion. All tissue digests were passed through sequential 70 and 40 μm cell strainers. Single cell suspensions were frozen in 10% DMSO/FCS using a Nalgene Mr. Frosty Freezing chamber (Thermo Fisher), before being transferred to liquid nitrogen for storage prior to use.

### Cell Sorting

Frozen endometrial cell digests or isolated PBMCs were thawed into HS-media (RPMI1640 + 10%FCS + 1% human serum + 2 mM glutamine + 100 IU/ml penicillin + 100 μg/ml streptomycin) at 37°C and centrifuged at 300 × *g*. Endometrial cells were re-suspended in HS-media and transferred to a 25 cm^2^ flask for 30 min at 37°C/5% CO_2_, then supernatants containing non-adhered cells were further transferred to a fresh flask for an additional 30 min incubation, for stromal and epithelial cell depletion. Supernatants were then removed lymphocytes isolated using lymphoprep (Axis-shield). Cells were washed in PBS and stained with DAPI (Biolegend, San Diego, CA, United States) and antibodies CD45 BV650^TM^, CD3 APC FIRE^TM^ 750, and CD19 PE (all from Biolegend, San Diego, CA, United States), then sorted using an Aria III (BD Biosciences, Franklin Lakes, NJ, United States) with 85 μm nozzle. CD19^+^ B cells were isolated as singlet cells (FSC-H vs FSC-A), Zombie Aqua^TM –^, CD45^+^, CD3^–^, CD19^+^. 6486 ± 7965 (mean ± SD) CD19^+^ B cells were sorted with sorting purity over 98% for all samples.

### B Cell Stimulation Assay

Sorted CD19^+^ B cells were placed in a 96 well U bottom plate (Eppendorf, Germany) with HS-media overnight, with <5,000 cells per well. Cells were then un-stimulated (controls) or treated with 1 mg/ml PMA + 1 mg/ml LPS + 1 mM ionomycin in 100 μl Culture Medium for 43 h, then Brefeldin A (Biolegend, San Diego, CA, United States) was added for an additional 5 h. Both stimulated and un-stimulated cells were then subjected to flow cytometric staining.

### RNA Extraction and Quantification

Immediately after sorting, cells were pelleted by centrifugation and re-suspended in RLT buffer (Qiagen, Germantown, MD, United States) and RNA was extracted using QIAGEN RNeasy Micro Kits (Qiagen, Germantown, MD, United States) according to the manufacturer’s protocol. RNA quantity and quality were accessed on Agilent 4200 TapeStation System (Agilent, Santa Clara, CA, United States) according to the manufacturer’s protocol.

### RNA Sequencing: Library Preparation, Sequencing and Data Analysis

The library was prepared using a Standard Nextera Illumina Library Prep kit using a unique dual- index strategy to barcode cDNA fragments. DNA samples were amplified for 16 cycles and the cDNA quantities assessed using a 2100 Bioanalyzer (Agilent, Santa Clara, CA, United States) with Quant-iT PicoGreen (Thermo Fisher, Waltham, MA, United States). Clustering and sequencing was carried out by Novogene Co., Ltd. Briefly, clustering of the index-coded samples was performed on a cBot Cluster Generation System using PE Cluster Kit cBot-HS (Illumina) and paired end RNA sequencing was performed using the Novoseq 6000 platform, with 125 bp/150 bp read length and approximately 25 million reads per sample. Quality control was performed by Novogene Co., Ltd and reads mapped to Ensemble Homo Sapiens cDNA database release 94. Data analysis was performed using R (version 3.5.3) and RStudio, heatmap of top 50 expressed genes in endometrial B cells was generated using DESeq2 (Love et al., 2014).

### Flow Cytometry Analysis

All flow cytometry reagents were from Biolegend unless otherwise stated. Fc receptors were blocked using FcR Blocking Reagent (Miltenyi, Germany). Samples were incubated with Zombie-Aqua Fixable Viability kit for 15 min in the dark, then cells washed in PBS/2%FCS and antibodies toward cell surface markers were added for 20 min at 4°C in the dark. Antibodies used were CD45-BV650^TM^ (HI30) or CD45-AlexaFluor700 (HI30), CD19-FITC (HIB19) or CD19- PECy7 (HIB19), CD27-PEDAZZLE^TM^ 594 (O323) or CD27-BV785^TM^ (O323), CD38-PE (HB-7) or CD38-APCFire^TM^ 750 (HB-7), CD24-APC (ML5) or CD24-BV421^TM^ (ML5), CD138-BV711^TM^ (MI15), IgM-PECy7 (MHM-88), IgG-BV421^TM^ (M1310G05), IgA-APCVio770 (REA1014, Miltenyi, Germany), CD83-BV785^TM^ (HB15e), CD74-PE (LN2), HLA DR-APC (L243), CD69-PE_DAZZLE^TM^ (FN50) or CD69-BV785^TM^ (FN50), CD3-APCFire^TM^ 750 (UCHT1), CD4-FITC (OKT4), ICOS-BV650^TM^ (C398.4A), CXCR5-BV421^TM^ (J252D4), PD1-APC (EH12.2H7), IgD-PE_DAZZLE^TM^ (IA6-2), FAS-BV711^TM^ (DX2). Afterward, cells were washed in PBS/2% FCS, and if necessary intracellular antibody staining was performed using Cytofix/Cytoperm Kit (BD, Franklin Lakes, NJ, United States), cells were fixed for 30 min then transferred into PBS/2% FCS overnight, then permeabilization performed before addition of antibodies IL-10-AlexaFluor488 (JES3-9D7), TGF-β-APC (TW4-6H10) or BCL6-PE (IG191E/A8) for 1 h incubation.

Data was acquired using an LSR-II flow cytometer (BD Biosciences) and data analyzed using FlowJo software (Tree Star Inc.), fluorescence Minus One (FMO) controls established gating strategies. Statistical analysis was performed using GraphPad Prism 7.03. Normal data distributions were confirmed using Shapiro-Wilk tests, parametric data were then analyzed using Student’s *t*-test. Data were represented as mean ± SD. Differences were considered significant if *p* < 0.05.

## Results

**FIGURE 1:**
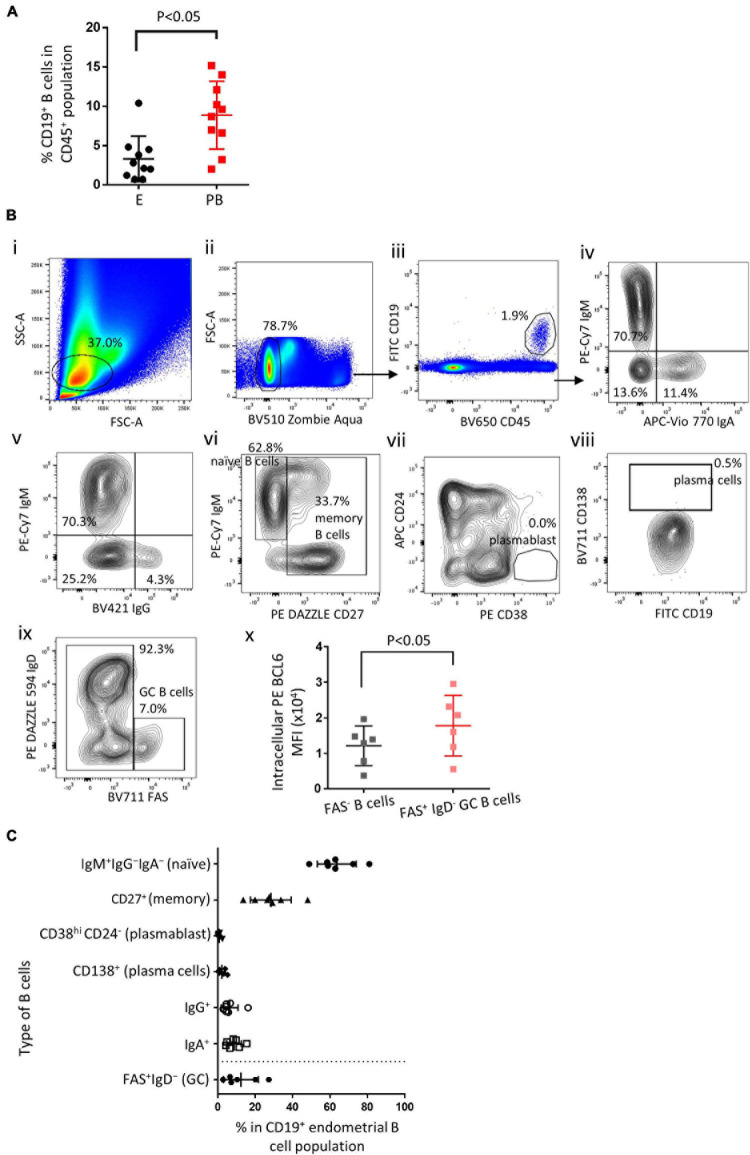
Endometrial B cell subsets analysis. **(A)** Flow cytometric analysis of B cells (*n* = 10) showing the proportion of B cells in the endometrium vs. peripheral blood. **(B)** Representative flow cytometric staining of endometrial B cells: i–iii, FSC-A vs. SSC-A and FSC-H determine lymphocyte singlet cells, ZA^lo^/CD45^+^/CD19^+^ were identified as B cells; iv and v, IgA^+^ and IgG^+^ B cells were identified; vi, IgM^+^CD27^–^ were identified as na ve B cells, CD27^+^ were identified as memory B cells; vii, CD24^–^ CD38^hi^ were identified as plasmablasts; viii, CD138^+^ were identified as plasma cells; ix, FAS^+^IgD^–^ were identified as GC B cells. x, Flow cytometric analysis of BCL6 MFI in GC B cells vs. FAS^–^ B cells (*n* = 6). **(C)** Flow cytometric analysis of the proportion of B cell subsets in the endometrium (*n* ≥ 6). Different flow cytometric panels were separated by a dash line. Dots are individual samples and bars = mean ± S.E.M.

Endometrial biopsies and peripheral blood samples were taken during the mid-luteal phase of the menstrual cycle. Single cell suspensions were obtained and flow cytometry was performed to identify endometrial B cells and peripheral blood B cells. Endometrial B cells (CD19^+^) comprised 3.3 ± 2.8% (mean ± SD) of the total CD45^+^ lymphocytes, which was significantly lower than found in the peripheral blood (Figure 1A, *n* = 10). Various B cell subset in the human endometrium were identified (representative flow cytometry staining Figure 1B(i-x) and summarized in Figure 1C, *n* ≥ 6). Endometrial immune cells were gated on FSC vs. SSC (1B-ii) and CD45^+^CD19^+^ events (1B-iii). The majority (63.6 ± 9.6%) of endometrial B cells were IgM^+^IgG^–^IgA^–^ and thus naïve B cells, 6.3 ± 4.1% and 8.6 ± 3.7% of all endometrial B cells were IgM^–^IgG^+^ and IgM^–^IgA^+^, respectively, (1B-iv and-v). 28.3 ± 10.1% of all endometrial B cells were CD27^+^ and therefore designated memory B cells (Figure 1B-vi). CD138^+^ plasma cells and CD38^hi^ CD24^–^ plasmablasts were scarce (<2%) in most endometrial B cell samples (Figure 1B-vii and -viii). In addition, FAS^+^IgD^–^ GC B cells were found in all six endometrial samples, accounting for 12.2 ± 8.6% of all CD19^+^ B cells (Figure 1B-ix), with a significantly higher level of intracellular BCL6 expression compared with FAS^–^ B cells (Figure 1B-x). Apart from GC B cells, which are largely absent in peripheral blood, the proportional distribution of endometrial B cell subsets is similar to that in the peripheral B cells, naïve B cells comprise about 60–70% of all B cells and memory B cells account for 20–30% of all B cells (Perez-Andres et al., 2010; Kaminski et al., 2012; Clavarino et al., 2016).

**FIGURE 2:**
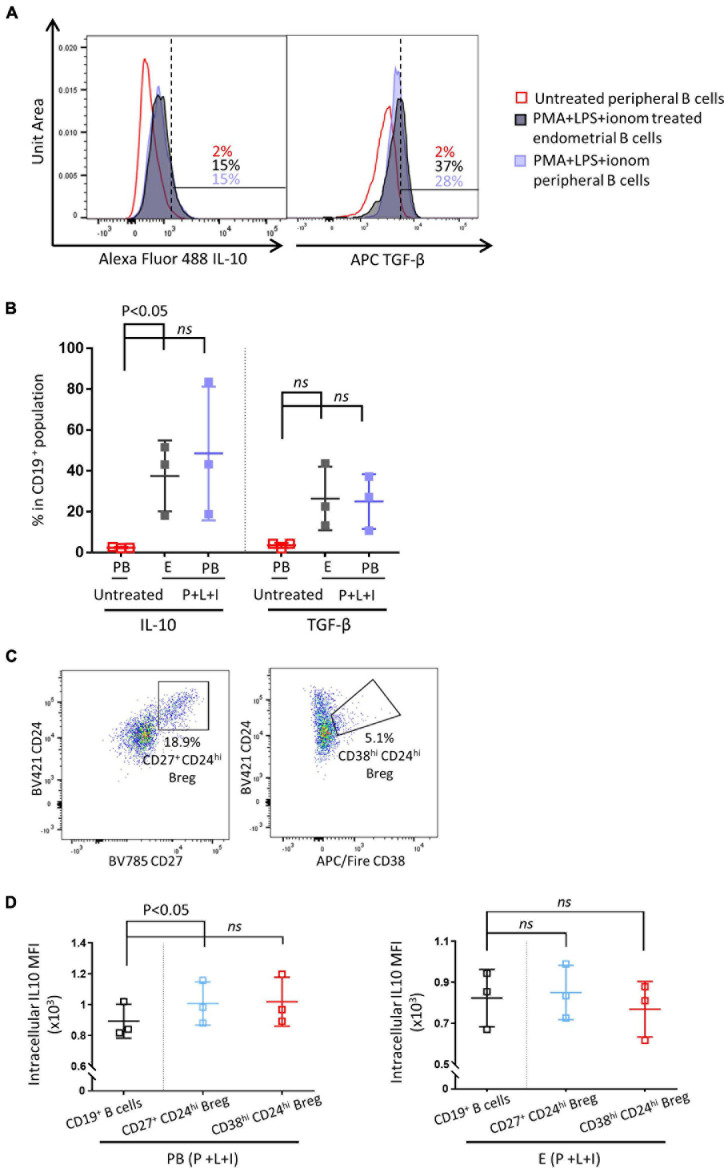
Endometrial and peripheral B cell IL-10 and TGF-β production after stimulation. Sorted endometrial and peripheral CD19^+^ B cells were either treated with PMS + LPS + ionomycin (P + L + I) or left untreated for 48 h. **(A)** Histograms of IL-10 and TGF-β expression levels in untreated peripheral B cells, P + I + L treated endometrial B cells and P + I + L treated peripheral B cells (*n* = 3). **(B)** Scatter plot of IL-10 and TGF-β expression levels in all groups. **(C)** Representative flow cytometric staining of endometrial Breg cells (CD24^hi^CD27^hi^ or CD24^hi^CD38^hi^). **(D)** Flow cytometric analysis of IL-10 MFI in peripheral and endometrial Breg cells vs. all B cells (*n* = 3). Dots are individual samples and bars = mean ± S.E.M.

Next, we examined whether IL-10 secreting Breg cells are present in the human endometrium. Peripheral and endometrial CD19^+^ B cells were obtained by flow cytometric cell sorting. Sorted CD19^+^ B cells were either stimulated with PMS + LPS + ionomycin, or left untreated for 48 h. As shown in Figures 2A,B, all peripheral and endometrial CD19^+^ B cells displayed a significantly higher level of IL-10 MFI after stimulation, the expression level of IL-10 was similar between simulated peripheral and endometrial CD19^+^ B cells (*n* = 3). They also exhibited a higher level of TGF-β after stimulation, but no significant difference was observed between stimulated and unstimulated groups (Figures 2,B, *p* = 0.06). In circulating CD19^+^ B cells, IL-10 production capacity has been reported to be enriched in the Breg populations, which can be identified by flow cytometry as CD27^+^CD24^hi^ or CD38^hi^CD24^hi^ (Hasan et al., 2019; Lin et al., 2019). In the peripheral blood samples, CD27^+^CD24^hi^ Bregs accounted for 10.6 ± 2.5% of all CD19^+^ B cells, CD38^hi^CD24^hi^ Bregs represented 6.2 ± 2.0% of all CD19^+^ B cells (*n* = 3). In the endometrial samples, CD27^+^CD24^hi^ Bregs were comprised 16.9 ± 0.9% of all CD19^+^ B cells, CD38^hi^CD24^hi^ Bregs comprised 11.0 ± 2.0% of all CD19^+^ B cells (Figure 2C, *n* = 3). As shown in Figure 2D, in peripheral CD19^+^ B cells, IL-10 expression was significantly enriched in CD27^+^CD24^hi^ Bregs, while the enrichment was not significant in CD38^hi^CD24^hi^ Bregs (*p* = 0.07). For endometrial CD19^+^ B cells, IL-10 expression was not enriched in either CD27^+^CD24^hi^ or CD38^hi^CD24^hi^ Breg populations (*p* = 0.1).

**FIGURE 3:**
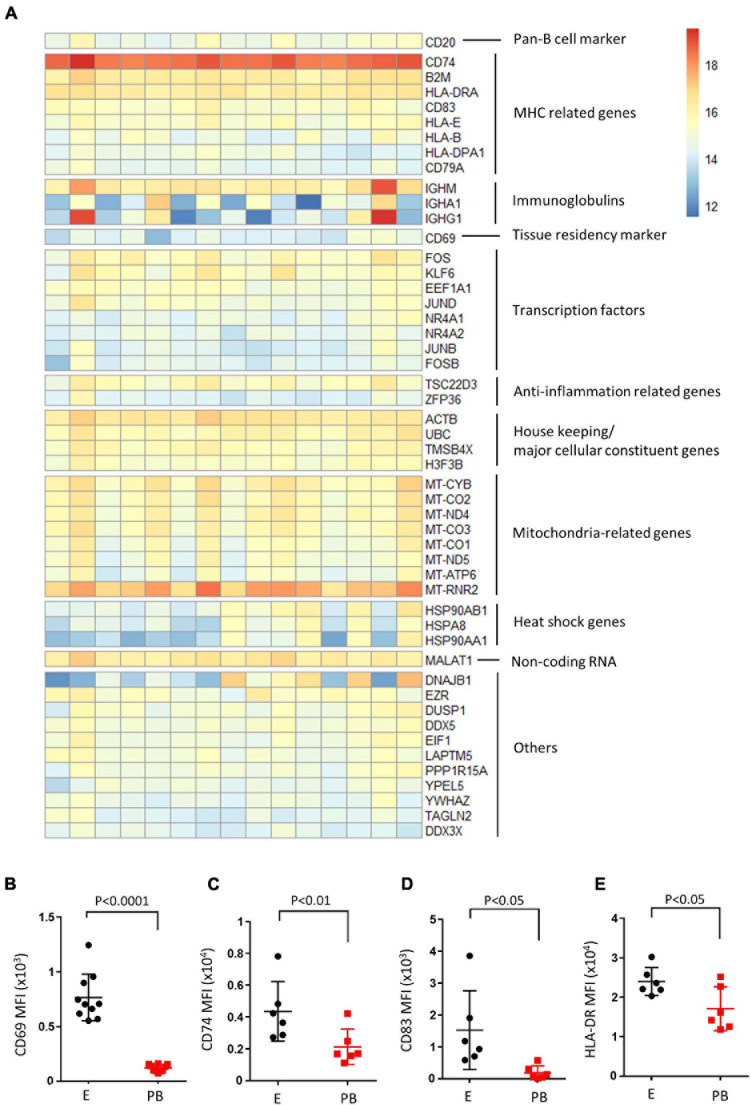
Endometrial B cells exhibit tissue resident-like phenotype with an activated MHC expression profile compared with peripheral B cells. **(A)** Heatmap of top 50 expressed genes in endometrial B cells. Genes were clustered based on their biological functions. **(B–E)** Flow cytometric analysis of B cells (*n* ≥ 6) showing the CD69 MFI, CD74 MFI, CD83 MFI, and HLA-DR MFI of CD19^+^ B cells in the endometrium vs. peripheral blood. Dots are individual samples and bars = mean ± S.E.M.

RNA-seq analysis on sorted CD19^+^ endometrial B cells was then performed on 15 samples. Overall, DESeq2 analysis returned 60,448 encoding genes, and 27,346 displayed non-zero read counts in the endometrial B cell sample set. The top 50 expressed genes from the endometrial B cell samples (*n* = 15) are illustrated, manually categorized into groups pertaining functions (Figure 3A). B cell signature genes were high, including pan-B cell marker (*CD20*), MHC related genes (*CD74*, *CD83*, *HLA-DR*, etc.) and immunoglobulin genes (*IGHM*, *IGHA1*, and *IGHG1*). Additionally, transcription factors (*FOS*, *KLF6*, *JUND*, etc.) and anti-inflammation related genes (*TSC22D3* and *ZFP36*) were highly expressed in endometrial B cell samples along with the tissue resident and early activation marker *CD69*. Housekeeping genes, mitochondrial functional related genes and heat shock genes are also highly expressed. Flow cytometry was then performed to examine the protein expression level of CD69, CD74, CD83, and HLA-DR on endometrial B cells compared to peripheral blood counterparts. We found endometrial B cells express significantly higher protein levels of CD69, CD74, CD83, and HLA-DR compared with peripheral B cells (*n* ≥ 6). This result suggests endometrial B cells have tissue resident-like phenotype with an activated MHC expression profile.

Having identified the presence of endometrial Fas^+^IgD^–^Bcl6^+^ GC-like B cells (Figure 1C), we also performed flow cytometry to detect if Tfh are present in the endometrium considering the functional importance of Tfh in GCs during B cell development. PD1^+^ICOS^+^CXCR5^+^ Tfh-like cells were identified in all 6 endometrial samples (Supplementary Figures 1A–C), they comprised 3.2 ± 1.6% of all CD4^+^ T cells. However, these Tfh-like cells did not have a higher level of intracellular BCL6 expression compared with PD1^–^ICOS^–^CD4^+^ T cells (Supplementary Figure 1A-iv). In terms of tissue residency, the majority of CD4^+^ T cells were CD69^+^, including PD1^+^ICOS^+^CXCR5^+^ Tfh-like cells and PD1^–^ICOS^–^CD4^+^ T cells (data not shown).

## Discussion

Endometrial B cells, largely consist of naïve B cells and memory B cells, have an activated phenotype compared with peripheral blood B cells, with a significant increase in CD74, HLA-DR, and CD83 expression levels. Both CD74 and HLA-DR have a well-established function in antigen presentation. CD74 is also known as the MHCII invariant chain that forms nonameric complexes with nascent MHCII molecules (Gil-Yarom et al., 2017), HLA-DR is an MHC class II cell surface receptor. The increased expression levels of CD74 and HLA-DR suggest an activated MHC class II signaling pathway in endometrial B cells, which could lead to tyrosine phosphorylation, calcium mobilization, AKT, ERK, and JNK activation (Mooney et al., 1990; Bouillon et al., 2003; Guo et al., 2003; Katikaneni and Jin, 2019). Studies have also shown that, in comparison to MHCII^–^ B cells, MHCII^+^ B cells had a substantial advantage in proliferation, differentiation into plasmablasts, or GC B cells, and isotype switching (Giles et al., 2015; Katikaneni and Jin, 2019). Similarly, CD83 has also shown to be a modulator for B cell maturation, activation and GC responses (Krzyzak et al., 2016; Li et al., 2019). Therefore, the upregulation of CD74, HLA-DR, and CD83 suggest endometrial B cells could undergo dynamic developmental processes such as GC reactions, and may playing an active role in the human endometrial immune environment. Additionally, an increased expression of tissue resident marker CD69 (Cibrián and Sánchez-Madrid, 2017; Savelyeva and Ottensmeier, 2020) is observed on endometrial B cells compared with their peripheral counterparts, confirmed that the majority of endometrial B cells are tissue resident-like B cells.

Flow cytometric analysis revealed the existence of GC B cells (CD19^+^FAS^+^IgD^–^BCL6^+^) and Tfh-like cells (CD4^+^PD1^+^ICOS^+^CXCR5^+^) in the human endometrium by flow cytometry. The tissue resident-like GC B cells indicated that endometrial B cells are likely to be an active player in the endometrial immune environments, possibly through GC reactions and the generation of endometrial memory B cells, which represent around 28% of all endometrial B cells. The presence of GC B cells correlates with the upregulation of CD74, HLA-DR, and CD83 on endometrial B cells. Higher levels of CD74 and HLA-DR expression have been shown in GC B cells compared with naïve and memory B cells, indicating the synthesis of new MHCII molecules that could enhance B cell antigen presentation capacities (Chalouni et al., 2003). CD83 is also expressed on GC B cells. In murine studies, CD83 is used as a marker for light zone B cells and might be involved in the antigen presentation to Tfh cells in the light zone (Krzyzak et al., 2016; Li et al., 2019). Apart from GC B cells, CD4^+^PD1^+^ICOS^+^CXCR5^+^ Tfh-like cells are also identified in the human endometrium, accounting for approximately 3.2% of all CD4^+^ T cells. However, compared with CD4^+^PD1^–^ICOS^–^CXCR5^–^ T cells, Tfh-like cells do not have a higher expression of BCL6, which is a defining marker for Tfh cells (Crotty, 2014). Although BCL6 expression levels fluctuate during Tfh cell development, Tfh cells should express higher level of BCL6 compared with CD4^+^PD1^–^ICOS^–^CXCR5^–^ T cells (Baumjohann et al., 2011; Li and Pauza, 2015). It might be possible that during the mid-luteal phase, the induction and initial development of GC-like reaction was formed independent of Tfh cells (Lentz and Manser, 2001). This GC-like reaction could later be developed into a Tfh-dependent GC, which may contribute to the generation of class-switched B cells and plasmablasts found in the decidua (Huang et al., 2017; Leng et al., 2019). Additionally, the infiltration of plasma cells has been described in endometrial inflammatory conditions such as endometritis (Cicinelli et al., 2019; Song et al., 2019; Zargar et al., 2020), there is a possibility that these GC B cells and Tfh-like cells are implicated in this process by providing the site of plasma cell generation. This proposed GC-like reaction could be linked to the LAs structure identified in the endometrial lower functional layer or the basal layer, where B cells are surrounded by T cells and macrophages (Klentzeris et al., 1992; Mettler et al., 1997; Yeaman et al., 1997, 2001; Disep et al., 2004). Further research is needed in understanding the cellular components and function of endometrial LAs and whether they can be classified as novel TLSs, and if they alter in endometrial associated reproductive pathologies. Our data are from a mixture of both fertile women and those with associated reproductive pathology, therefore it may be possible TLS are present in both healthy control women and those effected by disease.

Endometrial B cells are also capable of IL-10 production after stimulation, with a similar IL-10 expression level compared with matched peripheral blood samples. Considering the functional importance of IL-10 producing B cells in early pregnancy (Jensen et al., 2013; Guzman-Genuino and Diener, 2017; Esteve-Solé et al., 2018; Busse et al., 2019), this finding could help us to better understand the B cell role in the endometrial immune environment, and how different cellular components interact with each other.

## Data Availability Statement

The data presented in the study are deposited in the GEO repository, accession number GSE180728.

## Ethics Statement

The studies involving human participants were reviewed and approved by Oxford C Research Ethics Committee (ref:08/H0606/94) and (ref:18/SC/0216). The patients/participants provided their written informed consent to participate in this study.

## Author Contributions

MS designed the experiments, conducted the studies, analyzed the data, and wrote and edited the manuscript. JS and IG assisted in designing experiments and manuscript writing and editing. JS conceived the project and supervised the work. TC, MM, GS, and RS assisted with obtaining samples. All authors reviewed and approved the final version of the manuscript.

## Conflict of Interest

The authors declare that the research was conducted in the absence of any commercial or financial relationships that could be construed as a potential conflict of interest.

## Publisher’s Note

All claims expressed in this article are solely those of the authors and do not necessarily represent those of their affiliated organizations, or those of the publisher, the editors and the reviewers. Any product that may be evaluated in this article, or claim that may be made by its manufacturer, is not guaranteed or endorsed by the publisher.

## References

[B1] Baumjohann D. Okada T. Ansel K. M. (2011). Cutting edge: distinct waves of BCL6 expression during T follicular helper cell development. *J. Immunol.* 187 2089–2092. 10.4049/jimmunol.1101393 21804014

[B2] Bouillon M. El Fakhry Y. Girouard J. Khalil H. Thibodeau J. Mourad W. (2003). Lipid raft-dependent and -independent signaling through HLA-DR molecules. *J. Biol. Chem.* 278 7099–7107. 10.1074/jbc.M211566200 12499388

[B3] Brazdova A. Senechal H. Peltre G. Poncet P. (2016). Immune aspects of female infertility. *Int. J. Fertil. Steril.* 10 1–10. 27123194 10.22074/ijfs.2016.4762 PMC4845518

[B4] Busse M. Campe K.-N. J. Nowak D. Schumacher A. Plenagl S. Langwisch S. (2019). IL-10 producing B cells rescue mouse fetuses from inflammation-driven fetal death and are able to modulate T cell immune responses. *Sci. Rep.* 9:9335. 10.1038/s41598-019-45860-2 31249364 PMC6597542

[B5] Chalouni C. Banchereau J. Vogt A. B. Pascual V. Davoust J. (2003). Human germinal center B cells differ from naive and memory B cells by their aggregated MHC class II-rich compartments lacking HLA-DO. *Int. Immunol.* 15 457–466. 10.1093/intimm/dxg037 12663675

[B6] Cibrián D. Sánchez-Madrid F. (2017). CD69: from activation marker to metabolic gatekeeper. *Eur. J. Immunol.* 47 946–953. 10.1002/eji.201646837 28475283 PMC6485631

[B7] Cicinelli E. Bettocchi S. de Ziegler D. Loizzi V. Cormio G. Marinaccio M. (2019). Chronic endometritis, a common disease hidden behind endometrial polyps in premenopausal women: first evidence from a case-control study. *J. Minim. Invasive Gynecol.* 26 1346–1350. 10.1016/j.jmig.2019.01.012 30708117

[B8] Clavarino G. Delouche N. Vettier C. Laurin D. Pernollet M. Raskovalova T. (2016). Novel strategy for phenotypic characterization of human B lymphocytes from precursors to effector cells by flow cytometry. *PLoS One* 11:e0162209. 10.1371/journal.pone.0162209 27657694 PMC5033467

[B9] Crotty S. (2014). T follicular helper cell differentiation, function, and roles in disease. *Immunity* 41 529–542. 10.1016/j.immuni.2014.10.004 25367570 PMC4223692

[B10] Disep B. Innes B. A. Cochrane H. R. Tijani S. Bulmer J. N. (2004). Immunohistochemical characterization of endometrial leucocytes in endometritis. *Histopathology* 45 625–632. 10.1111/j.1365-2559.2004.02052.x 15569054

[B11] Esteve-Solé A. Luo Y. Vlagea A. Deyà-Martínez Á Yagüe J. Plaza-Martín A. M. (2018). B regulatory cells: players in pregnancy and early life. *Int. J. Mol. Sci.* 19:2099. 10.3390/ijms19072099 30029515 PMC6073150

[B12] Giles J. R. Kashgarian M. Koni P. A. Shlomchik M. J. (2015). B cell-specific MHC class II deletion reveals multiple nonredundant roles for B cell antigen presentation in murine lupus. *J. Immunol.* 195 2571–2579. 10.4049/jimmunol.1500792 26268653 PMC4561209

[B13] Gil-Yarom N. Radomir L. Sever L. Kramer M. P. Lewinsky H. Bornstein C. (2017). CD74 is a novel transcription regulator. *Proc. Natl. Acad. Sci. U. S. A.* 114 562–567. 10.1073/pnas.1612195114 28031488 PMC5255621

[B14] Guo W. Castaigne J.-G. Mooney N. Charron D. Al-Daccak R. (2003). Signaling through HLA-DR induces PKC beta-dependent B cell death outside rafts. *Eur. J. Immunol.* 33 928–938. 10.1002/eji.200323351 12672059

[B15] Guzman-Genuino R. M. Diener K. R. (2017). Regulatory B cells in pregnancy: lessons from autoimmunity, graft tolerance, and cancer. *Front. Immunol.* 8:172. 10.3389/fimmu.2017.00172 28261223 PMC5313489

[B16] Guzman-Genuino R. M. Eldi P. Garcia-Valtanen P. Hayball J. D. Diener K. R. (2019). Uterine B cells exhibit regulatory properties during the peri-implantation stage of murine pregnancy. *Front. Immunol.* 10:2899. 10.3389/fimmu.2019.02899 31921160 PMC6917594

[B17] Hasan M. M. Thompson-Snipes L. Klintmalm G. Demetris A. J. O’Leary J. Oh S. (2019). CD24hiCD38hi and CD24hiCD27+ human regulatory B cells display common and distinct functional characteristics. *J. Immunol.* 203 2110–2120. 10.4049/jimmunol.1900488 31511354

[B18] Huang B. Faucette A. N. Pawlitz M. D. Pei B. Goyert J. W. Zhou J. Z. (2017). Interleukin-33-induced expression of PIBF1 by decidual B cells protects against preterm labor. *Nat. Med.* 23 128–135. 10.1038/nm.4244 27918564 PMC5512431

[B19] Jensen F. Muzzio D. Soldati R. Fest S. Zenclussen A. C. (2013). Regulatory B10 cells restore pregnancy tolerance in a mouse model. *Biol. Reprod.* 89:90. 10.1095/biolreprod.113.110791 23986569

[B20] Kaminski D. A. Wei C. Qian Y. Rosenberg A. F. Sanz I. (2012). Advances in human B cell phenotypic profiling. *Front. Immunol.* 3:302. 10.3389/fimmu.2012.00302 23087687 PMC3467643

[B21] Katikaneni D. S. Jin L. (2019). B cell MHC class II signaling: a story of life and death. *Hum. Immunol.* 80 37–43. 10.1016/j.humimm.2018.04.013 29715484 PMC6207480

[B22] Klentzeris L. D. Bulmer J. N. Warren A. Morrison L. Li T. C. Cooke I. D. (1992). Endometrial lymphoid tissue in the timed endometrial biopsy: morphometric and immunohistochemical aspects. *Am. J. Obstet. Gynecol.* 167 667–674. 10.1016/s0002-9378(11)91568-3 1530020

[B23] Krzyzak L. Seitz C. Urbat A. Hutzler S. Ostalecki C. Gläsner J. (2016). CD83 modulates B cell activation and germinal center responses. *J. Immunol.* 196 3581–3594. 10.4049/jimmunol.1502163 26983787

[B24] LeBien T. W. Tedder T. F. (2008). B lymphocytes: how they develop and function. *Blood* 112 1570–1580. 10.1182/blood-2008-02-078071 18725575 PMC2518873

[B25] Lee K. M. Stott R. T. Zhao G. SooHoo J. Xiong W. Lian M. M. (2014). TGF-β-producing regulatory B cells induce regulatory T cells and promote transplantation tolerance. *Eur. J. Immunol.* 44 1728–1736. 10.1002/eji.201344062 24700192 PMC4048633

[B26] Leng Y. Romero R. Xu Y. Galaz J. Slutsky R. Arenas-Hernandez M. (2019). Are B cells altered in the decidua of women with preterm or term labor? *Am. J. Reprod. Immunol.* 81:e13102. 10.1111/aji.13102 30768818 PMC6556388

[B27] Lentz V. M. Manser T. (2001). Cutting edge: germinal centers can be induced in the absence of T cells. *J. Immunol.* 167 15–20. 10.4049/jimmunol.167.1.15 11418626

[B28] Li H. Pauza C. D. (2015). CD25(+) Bcl6(low) T follicular helper cells provide help to maturing B cells in germinal centers of human tonsil. *Eur. J. Immunol.* 45 298–308. 10.1002/eji.201444911 25263533 PMC4293275

[B29] Li Z. Ju X. Silveira P. A. Abadir E. Hsu W.-H. Hart D. N. J. (2019). CD83: activation marker for antigen presenting cells and its therapeutic potential. *Front. Immunol.* 10:1312. 10.3389/fimmu.2019.01312 31231400 PMC6568190

[B30] Lin X. Wang X. Xiao F. Ma K. Liu L. Wang X. (2019). IL-10-producing regulatory B cells restrain the T follicular helper cell response in primary Sjögren’s syndrome. *Cell. Mol. Immunol.* 16 921–931. 10.1038/s41423-019-0227-z 30948793 PMC6884445

[B31] Love M. I. Huber W. Anders S. (2014). Moderated estimation of fold change and dispersion for RNA-seq data with DESeq2. *Genome Biol.* 15:550. 10.1186/s13059-014-0550-8 25516281 PMC4302049

[B32] Lucas E. S. Vrljicak P. Muter J. Diniz-da-Costa M. M. Brighton P. J. Kong C.-S. (2020). Recurrent pregnancy loss is associated with a pro-senescent decidual response during the peri-implantation window. *Commun. Biol.* 3:37. 10.1038/s42003-020-0763-1 31965050 PMC6972755

[B33] Mettler L. Jürgensen A. Volkov N. I. Kulakov V. Parwaresch M. R. (1997). lmmuno histochemical profile of endometrium in patients with genital endometriosis. *Diagn. Ther. Endosc.* 3 127–145. 10.1155/DTE.3.127 18493428 PMC2362564

[B34] Mooney N. A. Grillot-Courvalin C. Hivroz C. Ju L. Y. Charron D. (1990). Early biochemical events after MHC class II-mediated signaling on human B lymphocytes. *J. Immunol.* 145 2070–2076. 2398273

[B35] Perez-Andres M. Paiva B. Nieto W. G. Caraux A. Schmitz A. Almeida J. (2010). Human peripheral blood B-cell compartments: a crossroad in B-cell traffic. *Cytometry B Clin. Cytom.* 78(Suppl. 1) S47–S60. 10.1002/cyto.b.20547 20839338

[B36] Pipi E. Nayar S. Gardner D. H. Colafrancesco S. Smith C. Barone F. (2018). Tertiary lymphoid structures: autoimmunity goes local. *Front. Immunol.* 9:1952. 10.3389/fimmu.2018.01952 30258435 PMC6143705

[B37] Savelyeva N. Ottensmeier C. H. (2020). Novel players: tissue-resident memory B cells. *Blood* 136 2722–2723. 10.1182/blood.2020007890 33301032

[B38] Song D. Li T.-C. Zhang Y. Feng X. Xia E. Huang X. (2019). Correlation between hysteroscopy findings and chronic endometritis. *Fertil. Steril.* 111 772–779. 10.1016/j.fertnstert.2018.12.007 30683588

[B39] Vajdy M. (2006). Generation and maintenance of mucosal memory B cell responses? *Curr. Med. Chem.* 13 3023–3037. 10.2174/092986706778521760 17073644

[B40] Vallvé-Juanico J. Houshdaran S. Giudice L. C. (2019). The endometrial immune environment of women with endometriosis. *Hum. Reprod. Update* 25 564–591. 10.1093/humupd/dmz018 31424502 PMC6737540

[B41] Wira C. R. Rodriguez-Garcia M. Patel M. V. (2015). The role of sex hormones in immune protection of the female reproductive tract. *Nat. Rev. Immunol.* 15 217–230. 10.1038/nri3819 25743222 PMC4716657

[B42] Yeaman G. R. Collins J. E. Fanger M. W. Wira C. R. Lydyard P. M. (2001). CD8+ T cells in human uterine endometrial lymphoid aggregates: evidence for accumulation of cells by trafficking. *Immunology* 102 434–440. 10.1046/j.1365-2567.2001.01199.x 11328377 PMC1783206

[B43] Yeaman G. R. Guyre P. M. Fanger M. W. Collins J. E. White H. D. Rathbun W. (1997). Unique CD8+ T cell-rich lymphoid aggregates in human uterine endometrium. *J. Leukoc. Biol.* 61 427–435. 9103229

[B44] Zargar M. Ghafourian M. Nikbakht R. Mir Hosseini V. Moradi Choghakabodi P. (2020). Evaluating chronic endometritis in women with recurrent implantation failure and recurrent pregnancy loss by hysteroscopy and immunohistochemistry. *J. Minim. Invasive Gynecol.* 27 116–121. 10.1016/j.jmig.2019.02.016 30851430

