# Supplementary material for: B Cell Subset Analysis and Gene Expression Characterization in Mid-Luteal Endometrium
Source: Front Cell Dev Biol. 2021 Aug 10;9:709280. doi: 10.3389/fcell.2021.709280 (PMC8383145; doi:10.3389/fcell.2021.709280)
Supplement: Supplementary file 1 [file Data_Sheet_1.DOCX]

Supplementary Material

# Supplementary Figures


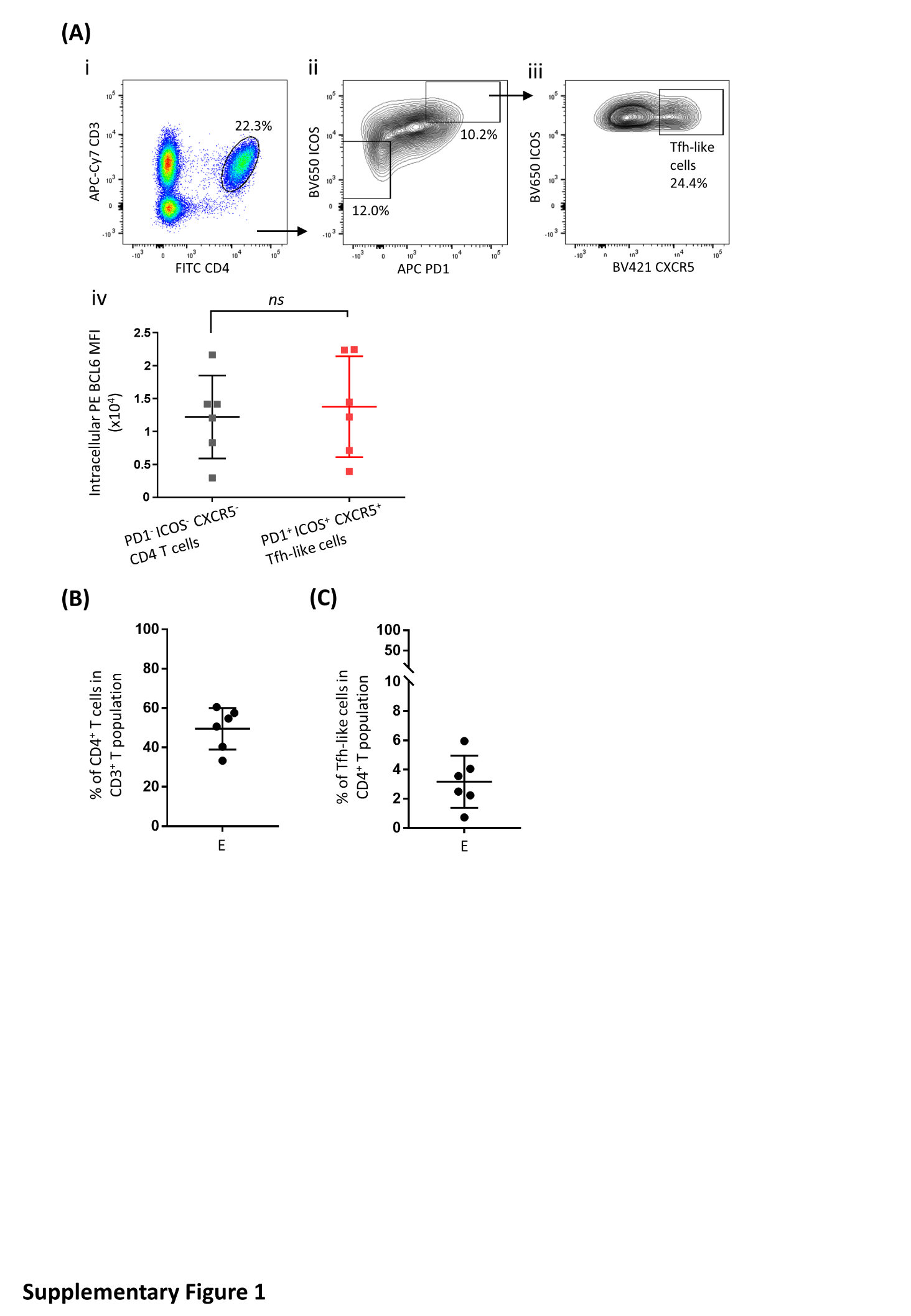


**Supplementary Figure 1.** Identification of endometrial Tfh-like cells. (A) i-iii, Representative flow cytometric staining of endometrial Tfh-like cells (CD4^+^PD1^+^ICOS^+^CXCR5^+^). iv, Flow cytometric analysis of BCL6 MFI in Tfh-like cells versus PD1^-^ICOS^-^CXCR5^-^ CD4 T cells (n=6). (B), The proportion of endometrial CD4^+^ T cells in CD3^+^T cells. (C) The proportion of endometrial Tfh-like cells in CD4^+^T cells. Dots are individual samples and bars = mean+/-S.E.M
