# Supplementary figures and images for: B Cell Subset Analysis and Gene Expression Characterization in Mid-Luteal Endometrium
Source: Front Cell Dev Biol. 2021 Aug 10;9:709280. doi: 10.3389/fcell.2021.709280 (PMC8383145; doi:10.3389/fcell.2021.709280)

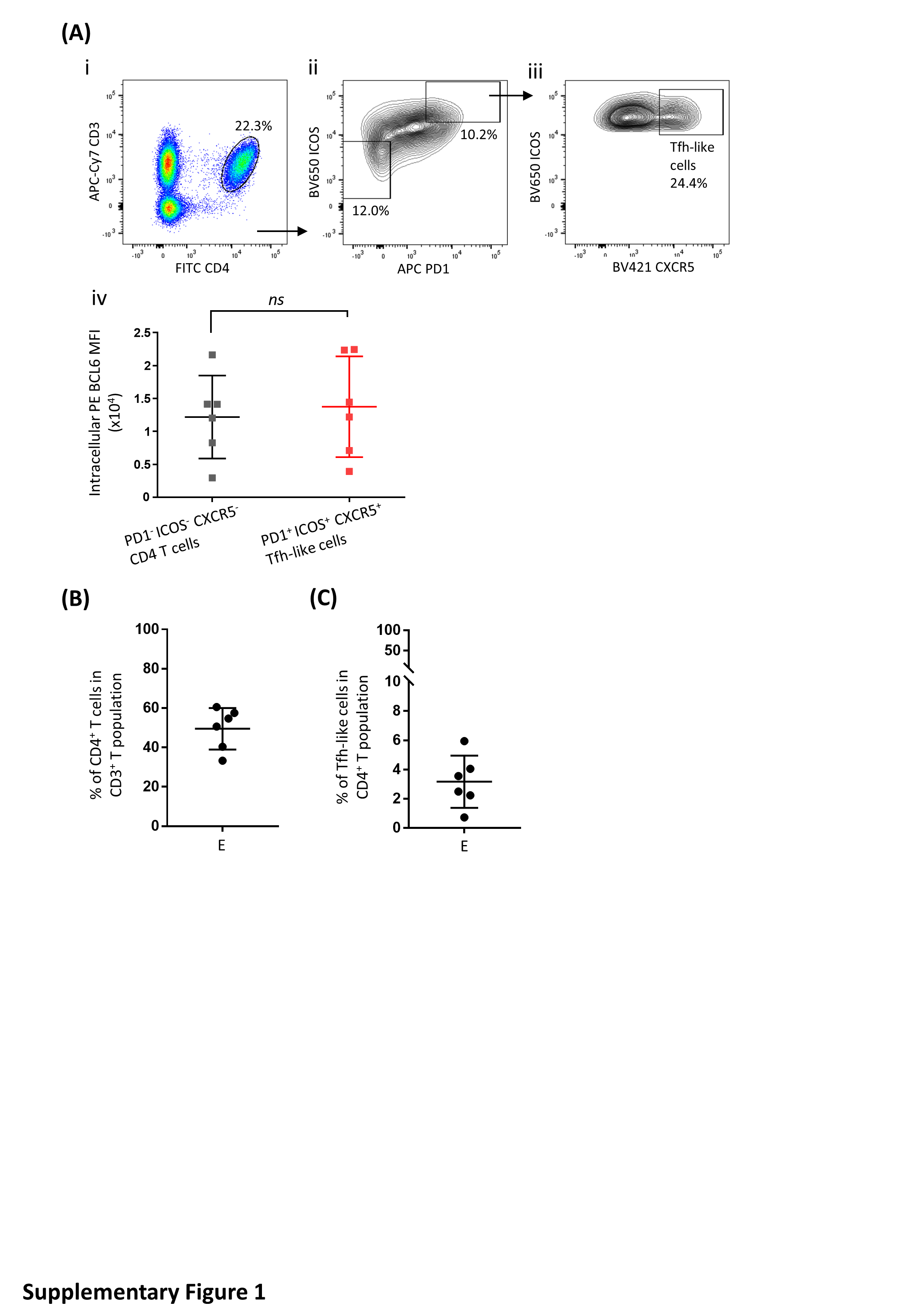

Supplement: Supplementary file 2 [file Image_1.TIF]
